# Exposing health inequities: Surreal snapshots from the Grand Canyon to global COVID-19 pandemic

**DOI:** 10.1177/1473325020973312

**Published:** 2021-03

**Authors:** Jean E Balestrery

**Affiliations:** Spirit of EAGLES, Rochester, USA

**Keywords:** Social work practice, crisis intervention, indigenous, Covid-19, health inequity, indigenous health

## Abstract

The story presented here is central to social work because it is about crisis. Across diverse fields of practice, social workers regularly engage in crisis intervention. The story that follows is about crisis in the area of health and healthcare. Specifically, it’s about exposing health/care inequities on Indigenous tribal land in the Grand Canyon and in the global COVID-19 pandemic.

## Trail map

Circuitous is the word that best describes my career trajectory in social work. What this means is I began my career in practice, then spent many years in research, followed by years in teaching and later back again in practice. Currently, I do some of each.

Traversing these professional switchbacks situates me as a scholar-practitioner. With each step, my horizon expands and reveals gaps in care. These gaps are seen through my interdisciplinary lens in social work and anthropology, in addition to my LGBTTQ+, White, Euro-American, cisgender female and formally educated perspectives. I am an applied anthropologist and peripatetic practitioner endeavoring to close the health gap – a Social Work Grand Challenge.

The story presented here is central to social work because it is about crisis. Across diverse fields of practice, social workers regularly engage in crisis intervention. The story that follows is about crisis in the area of health and healthcare. Specifically, it’s about exposing health/care inequities on Indigenous tribal land in the Grand Canyon and amidst the global COVID-19 pandemic.

## Breaking news

We are experiencing a global pandemic. Here in the United States (U.S.), we are under national orders to practice “social distancing,” or physical distancing, and many of us are under additional state orders to shelter in place: stay inside to prevent the spread of COVID-19 and save lives - literally save lives. These are unprecedented times on a global scale. In the U.S., we are repeatedly hearing that “we are all in this together.” Our current lived reality is described as The Great Adaptation, due to the current global public health crisis. How we respond matters.

## Reflection

My mind replays all that could have happened: could’ve drowned, could’ve hit my head, could’ve lost my foot, could’ve bled to death, could’ve lost consciousness, could’ve crashed – could’ve, could’ve, could’ve - but didn’t. None of these things happened, but most certainly could have happened. The therapist I visited for a few months after the accident told me during my initial session: “you got hit by a spiritual two-by-four …” I was flummoxed. During the initial post-accident months, I cycled through a roller-coaster of emotions: feeling scared, powerless, confused, anxious, irritable, angry, sad, *and* grateful.

These sessions combined with physical therapy aided my post-traumatic growth following a near-death hiking accident in the Grand Canyon. For years I had been wanting to visit Havasu Falls on Havasupai tribal lands in the Grand Canyon. The eight-mile hike into -and back out from- this international tourist destination did not deter me (Collins, 2018a, 2018b, 2018c).

I’ll never forget the impact of seeing Native Elders with walking sticks appear in front of me as I lay on a stretcher, at the top of Havasu Falls trail. I locked eyes with one of the Native Elders, then signaled “thumbs up” with my right hand and smiled as tears rolled down my face. He signaled “thumbs up” in return, then looked at my hiking friend and said: “We call that area … ” -turning his head and pointing to the waterfalls area where I had fallen - “meat grinder.”

## Breaking news

We are experiencing a global pandemic. It is surreal: the number of coronavirus cases and deaths increases daily across the globe. Information about this grim reality is never-ending and various news headlines read: “Coronavirus Pandemic ‘Is a Call to Action,’ U.N. Secretary General Says” and “The United Nations issued a report calling the coronavirus the most challenging issue the world has faced since World War Two” (Reuters, 2020). We are in a war with an invisible enemy - a virus, and the U.S. is now building field hospitals. How we respond matters.

## The accident

The rushing water over the boulder rock face that I climbed onto was only a foot high. Yet, as soon as I had all fours on the rock face, on my way to the other side of the waterfall pool, a tsunami force took hostage of me! Luckily, the hand-holds I grabbed were deep enough to hang onto while the raging water whiplashed my face. I gulped in air as much as I could as I hung on, then *boom!* The waterfall current hurled my body down many feet and slammed it into the rock bottom of the waterfall pool below.

Instinctively thrashing my arms, I saw shimmers of light above me then swam upwards to air. A surreal snapshot: *that was my life*! resounded within me as blood swirled in the water. I turned my head to locate the river bank and saw in the distance my hiking friend running.

“Grab my arm! Grab my arm!” my hiking friend yelled. After jointly getting my body up and over the three-foot river bank edge, we both saw my left foot dangling outward from my ankle at about a 90-degree angle: a disturbing sight of deformity. My ankle was cut – or sliced – and bleeding; later, I learned it was dislocated and that I had a severe compound fracture. My friend, a professional backpacking guide, hadn’t ever experienced an accident while leading dozens of trips to this tourist destination. Ironically, I had to direct him to go get help.

## Breaking news

COVID-19 data reveal growing hostilities and inequities (Centers for Disease Control and Prevention, 2020; Flitter, 2020; Godoy and Wood, 2020; Hooper et al., 2020; van Dorn et al., 2020): across the U.S., hate crimes against Asian American people have increased (Hong, 2020; Shen-Berro, 2020); Black people comprise 13% of the population in the U.S., yet 24% of COVID-19 deaths where race is known (Abassi, 2020); Black people have a COVID-19 mortality rate that is more than double that of any other racial/ethnic group in the U.S. (Caruso, 2020); the Navajo Nation had more confirmed COVID-19 cases per-capita than every state in the U.S. (Silverman et al., 2020). We are all in the same storm, yet each of us is in a different boat. How we respond matters.

## Fight, flight or freeze

It took approximately five and a half hours to get out of the Grand Canyon. When the tribal clinic doctor finally arrived at Havasu Falls trailhead - where impromptu helpers carried me, he told me “I’m not going to lie, it’s going to be hell riding back up the trail … ” Lying in a metal stretcher hanging off the end of an ATV, I prayed my foot wouldn’t fall off.

I asked the doctor what hospital I’d be going to and he said “most likely Kingman” – it was closest. I kindly let him know that I preferred to go to Flagstaff because I knew *that* hospital would be able to handle my injury. My years of working in rural and remote village Alaska -with Indigenous peoples on tribal land- taught me that rural healthcare services are typically limited.

At the tribal clinic, the doctor told me I might need to stay overnight because helicopter companies weren’t flying due to bad weather. Hiking out the eight miles was not an option. In an instant, I sprung up in the clinic bed and with a self-determined force said to the doctor: “*I have got to get to Flagstaff Medical Center!”* This act of advocacy no doubt saved my foot. The social worker in me knew it and my medical team confirmed it: “it could have been catastrophic.” The doctor made more calls to get a rescue helicopter, which flew me direct to the Flagstaff hospital.

## Breaking news

Many months into this global pandemic crisis, we are reminded to *#stay home* – yet not everyone can - and *#stay safe* - yet, not everyone is. With an outpouring of community support and gratitude for front line and essential workers, there is also foreshadowing of an economic crisis and mental health crisis – both are looming. A COVID-19 vaccine is many months away. Social Work: Our current call to action is *#remaining resilient.* Social work, the times we are in is the legacy of our profession (Golden et al., 2020). *#be brave, #be bold.* How we respond matters.

## Living gaps in care

Gap 1: *Missing Emergency Protocol.* I was shocked to discover there was no emergency protocol at this international tourist destination on tribal land. As I lay on a stretcher at the top of Havasu Falls trail, with my impromptu helpers brainstorming my exit, a tribal campground ranger arrived at the scene. I saw my hiking friend approach this ranger and ask: “What is your protocol in an emergency like this?”; the ranger then said “we don’t have one.”

Gap 2: *Missing Medical Attention.* I was shocked to find myself completely alone in the tribal clinic for about 20 minutes as I lay on the (only) bed. “hello? … hello? … is anyone Here? … Hello?? … HELLO????” No response. I needed help to get to the bathroom, and later wondered what might’ve happened if I had fallen unconscious. The researcher in me fixated on the wall clock, watching the hands mark each eternal minute. Later, I learned that everyone in the clinic went outside to meet the helicopter as it landed.

Gap 3: *Missing Social Worker.* During the arduous journey of approximately five and a half hours to get out of the Grand Canyon, there was no social worker. Hence, there was no leader to manage the overall crisis response, no advocate for my best interest, no coordinator for my care needs, no broker of resources and nobody to provide the emotional support and psychological first aid critical to a person’s felt sense of safety during a crisis.

## Breaking news

The U.S. government passes the CARES Act for economic aid, yet it ignites tension - between tribes and the U.S. government *and* among tribes. Congress allocates billions in funding relief to tribal governments, including the 12 regional Alaska Native for-profit corporations generating more than $10.5 billion in revenues in 2018. The Navajo Nation joins other tribes in a lawsuit against U.S. Secretary of Treasury advocating that funding go to tribes, not corporation shareholders (The Navajo Nation, 2020). *#overcoming these divisions #it takes all of us* (Balestrery et al., 2020) How we respond matters.

## The recovery process

“It’s your training and background, you knew what to do - most people wouldn’t have,” my friend explained to me as I awaited my next surgery. Listening to my story, she said in a definitive tone: “You managed your own crisis.” No doubt my background helped: I had researched Indigenous health disparities on and off tribal lands, taught crisis intervention courses and as a practitioner had helped many other people in crisis.

In total, I had three surgeries: two to prevent infection and one to reconstruct my ankle. Once I was back home recovering, I googled “accidents at Havasu Falls” and discovered there had been numerous accidents - and deaths - over many years on Havasupai tribal land. This was disturbing to learn, particularly within context of the reality that about 25,000 tourists a year visit Havasupai tribal lands. Further, the tribal tourism business was yielding millions of dollars in annual revenue for the Havasupai tribe yet all that was visible on the tribal land was stark poverty (McGivney, 2017).

My recovery regimen was all-consuming. The initial non-weight bearing phase was about three and a half months. However, I began daily physical therapy exercises every hour on the hour as soon as a couple weeks after my surgeries. Next, I attended physical therapy sessions regularly at an office location. These sessions pushed my physical limits and helped improve my mental stamina. My other option: a club foot. Once I could bear weight, I felt alive and free!

## Breaking news

On May 25th, George Floyd dies after being pinned for eight minutes forty-six seconds under the knee of a White male police officer on a street in Minneapolis, Minnesota. We are now experiencing another pandemic: protests igniting against police brutality. All are rising: COVID-19 cases and deaths, Black Lives Matter voices and unemployment rates – along with discourse on systemic and structural racism (Stolberg, 2020). The current pandemics are impacting everyone, either directly or indirectly through collateral consequences. Collective Futures: *#healing trauma #rethinking #reimagining #restructuring* How we respond matters.
